# Evaluation of the efficacy of ivermectin against *Theileria orientalis* infection in grazing cattle

**DOI:** 10.1186/s12917-019-2042-2

**Published:** 2019-08-17

**Authors:** Jinho Park, Jeong-Byoung Chae, Suhee Kim, Do-Hyeon Yu, Hyeon-Cheol Kim, Bae-Keun Park, Joon-Seok Chae, Kyoung-Seong Choi

**Affiliations:** 1 College of Veterinary Medicine, Chonbuk National University, Iksan, 54596 South Korea; 2 Laboratory of Veterinary Internal Medicine, BK21 PLUS Program for Creative Veterinary Science Research, Research Institute for Veterinary Science and College of Veterinary Medicine, Seoul National University, Seoul, 08826 South Korea; 3 Gyeongsang National University Hospital, Jinju, 52727 South Korea; 4 Institute of Animal Medicine, College of Veterinary Medicine, Gyeongsang National University, Jinju, 52828 South Korea; 5 College of Veterinary Medicine, Kangwon National University, Chuncheon, 24341 South Korea; 6 College of Veterinary Medicine, Chungnam National University, Daejeon, 34134 South Korea; 7 College of Ecology and Environmental Science, Kyungpook National University, Sangju, 37224 South Korea

**Keywords:** Grazing, Hematocrit, Ivermectin, *Theileria orientalis*

## Abstract

**Background:**

Raising cattle on pastures is known to be beneficial for animal welfare and cost reduction. However, grazing is associated with the risk of contracting tick-borne diseases, such as theileriosis. Here, the efficacy of ivermectin against these diseases and associated clinical symptoms were evaluated.

**Results:**

A total of 68 cattle from a grazing cattle farm were selected and divided into two groups: the control group (17 cattle) with no preventive treatment and the ivermectin-treated group (51 cattle) in which cattle were treated with pour-on ivermectin prior to grazing. The infection rates of *Theileria orientalis* and the red blood cell (RBC) profile (e.g., RBC count, hematocrit value, and hemoglobin concentration) were compared in the spring (before grazing) and summer (during grazing) between the two groups. Based on PCR amplification of the major piroplasm surface protein (MPSP) gene, 12 cattle were positive for *T. orientalis* infection. Phylogenetic analysis revealed that the isolates identified in this study consisted of three MPSP types (1, 2, and 7). The *T. orientalis* infection rate in the control group during grazing was 3-fold higher than that in the ivermectin-treated group. Moreover, differences in RBC parameters during grazing were greater in the control group than in the ivermectin-treated group. In particular, the hematocrit value was significantly reduced in the control group.

**Conclusions:**

The results of this study demonstrated that ivermectin had protective effects against *T. orientalis* infection and RBC hemolysis in grazing cattle.

## Background

*Theileria orientalis* is a tick-borne hemoprotozoan parasite that can cause clinical disease and lead to significant economic losses in the livestock industry in the Asia-Pacific region through anemia, jaundice, growth retardation, and reduced body weight in cattle [1–3]. *T. orientalis* is transmitted to the host through blood-sucking infected hard ticks [4]. One of the vectors of *T. orientalis* is *Haemaphysalis longicornis*, which is the most commonly found tick in the Republic of Korea (ROK) [5, 6]. Anemia and abortion are common clinical outcomes of this disease [7, 8]. The importance of controlling *T. orientalis* infection has been highlighted by recent outbreaks reported in Australia and New Zealand [1, 9]. Recently, the prevalence of *T. orientalis* infection has gradually increased in the ROK, and several genotypes *T. orientalis* have been reported [10]. However, no effective drugs or vaccines are currently available for controlling *T. orientalis*.

In comparison with conventional indoor housing, raising cattle on pastures is known to be beneficial for the health of animals and has many other advantages, including greater cattle activity, improvements in productivity, good animal welfare, and reduction in rearing costs and workloads [11, 12]. However, raising cattle on pastures may also have disadvantages, such as the risk of being bitten by ticks and developing infections through tick-borne pathogens with related clinical symptoms [13]. To prevent ticks and tick-borne diseases while maintaining the advantages of pasturing, several methods to control ticks and tick-borne pathogens have been developed and evaluated [14, 15]. Recently, the number of cattle grazing on pastures in the ROK has increased. Although many studies have investigated treatment approaches to prevent the infestation of ticks or methods that focus on the detection of pathogens after the application of anti-ectoparasitic chemicals, little is known about the effects of controlling tick-borne pathogens and related clinical symptoms in the host.

Among the various anti-ectoparasitic chemicals reported thus far, ivermectin is a useful drug for the management of grazing cattle [14, 16], and it is a well-known repellent for various ticks [17–20]. However, its effects on *H. longicornis* have not been evaluated. Therefore, the aim of this study was to evaluate the efficacy of ivermectin against *T. orientalis* infection and red blood cell (RBC) hemolysis by administration to cattle before grazing. This study may contribute to the development of control measures for bovine theileriosis.

## Results

**Fig. 1:**
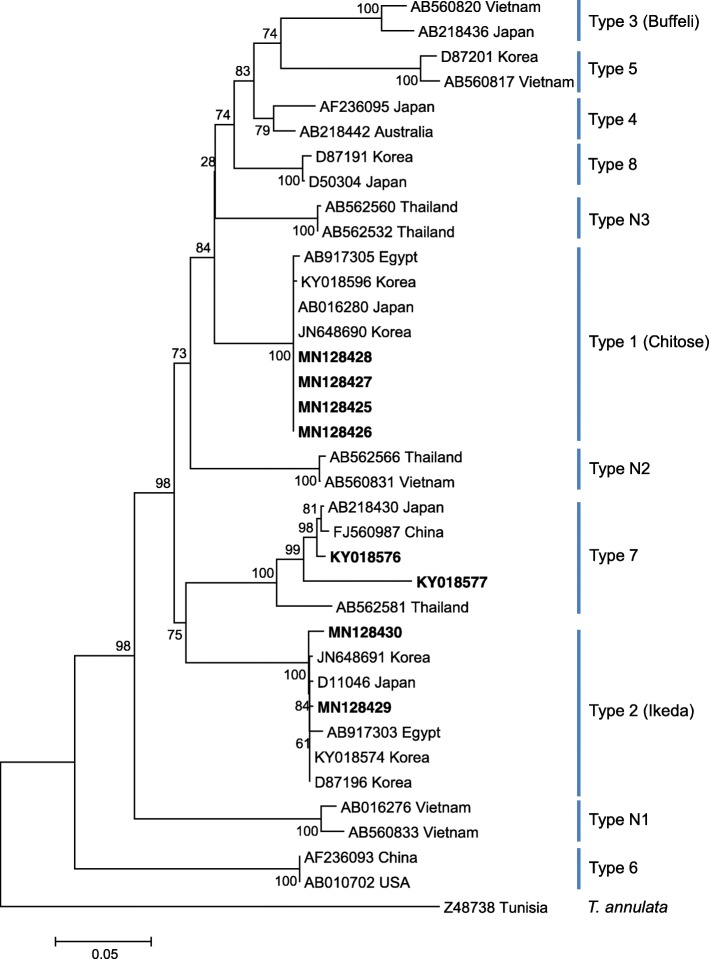
Phylogenetic analysis of *Theileria orientalis* based on the MPSP gene. A phylogenetic tree was constructed using MEGA7 software and the maximum likelihood method; numbers over branches indicate the bootstrap values in percentages (1000 replicates) that support each phylogenetic branch. The bold-faced type indicates sequences determined in this study

Of the tick-borne pathogens examined, only *T. orientalis* was detected in 12 cattle, and other tick-borne pathogens were not found in these cattle. These *T. orientalis*-positive cattle did not exhibit clinical symptoms, such as anorexia, depression, and reduced milk production. Based on the sequencing of all 12 amplicons, 8 good sequences were obtained and classified into three genotypes (4 sequences for type 1, 2 sequences for type 2, and 2 sequences for type 7) by phylogenetic analysis (Fig. 1). Four isolates, which belonged to type 1 (Chitose), showed 99.8–100% identity to each other, and these isolates were closely related to a Korean isolate (JN648690). Two isolates, which belonged to type 2 (Ikeda), showed 99.5–99.7% homology to a Korean isolate (KY018574) previously reported by our group. The nucleotide sequence similarity of the two isolates belonging to type 7 was 93.5% and these isolates formed the same clade with a Japanese isolate (AB218430) and a Chinese isolate (FJ560987) (Fig. 1). There was no mixture of genotypes in this herd.

**Fig. 2:**
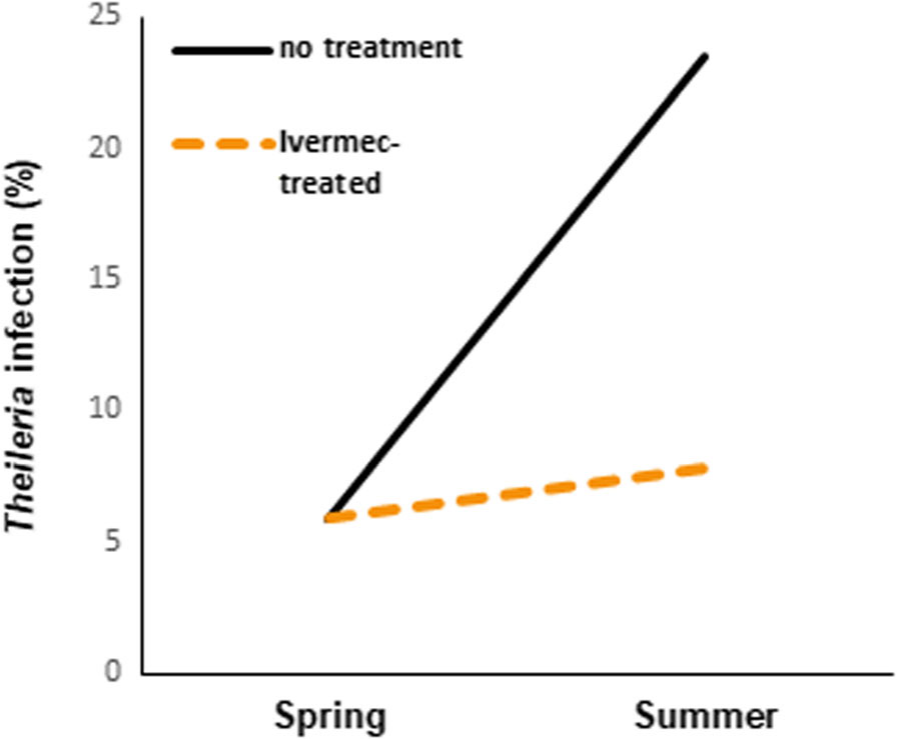
Comparison of the infection rates of *Theileria orientalis* between the control and ivermectin-treated groups in the spring and summer

To evaluate the efficacy of ivermectin, the infection rate of *T. orientalis* was compared between the control and ivermectin-treated groups. As shown in Fig. 2, in the control group, the infection rate of *T. orientalis* was 5.9% (1/17) in the spring (before grazing) and 23.5% (4/17) in the summer (during grazing). In contrast, in the ivermectin-treated group, the infection rate of *T. orientalis* was 5.9% (3/51) in the spring and 7.8% (4/51) in the summer. The infection rate of *T. orientalis* in both the control and ivermectin-treated groups during grazing was increased by 3.9-fold (23.5/5.9) and 1.3-fold (7.8/5.9), respectively, compared with the rate before grazing (Fig. 2). *T. orientalis* infection in the control group was increased by 3-fold compared with that in the ivermectin-treated group in the summer.

**Fig. 3:**
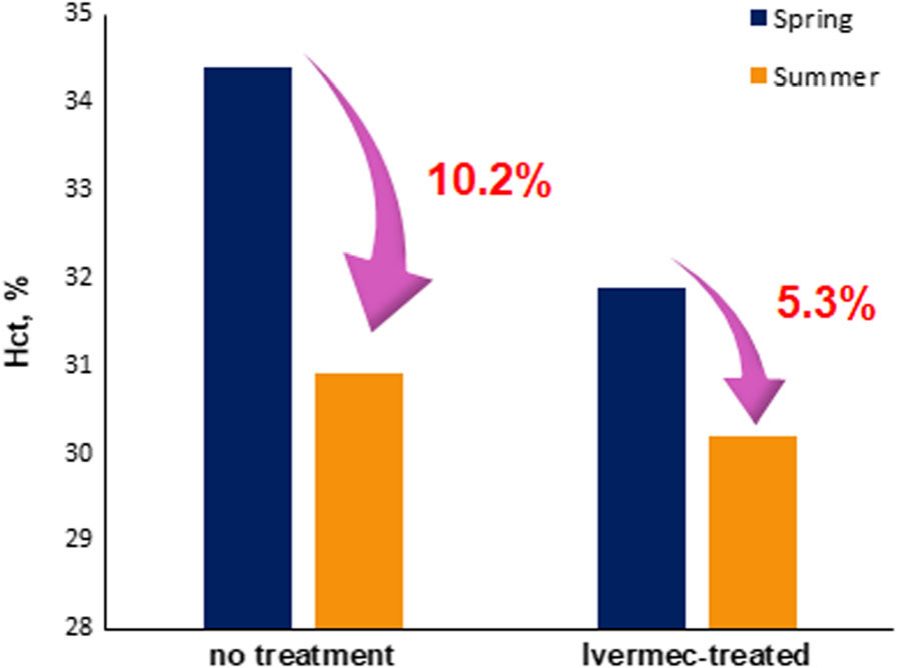
Differences in blood test results from spring to summer between the control and ivermectin-treated groups. Hematocrit (Hct) value was more reduced in control group than in ivermectin-treated group

Differences in blood test results from spring to summer between the control and ivermectin-treated groups were compared (Table 1). Seasonal differences in the RBC count (M/μL) and Hb concentration (g/dL) were lower in the control group than in the ivermectin-treated group; however, these results were not statistically significant. The average RBC count (M/μL), Hct value (%), and Hb concentration (g/dL) in the ivermectin-treated group were significantly decreased (*p* < 0.01) in the summer (Table 1). The largest difference was observed for the Hct value. In the control group, the Hct value was 34.4% in the spring and was reduced to 30.9% in the summer (10.2% reduction); in contrast, in the ivermectin-treated group, the Hct value was 31.9% before grazing and was decreased to 30.2% during grazing (5.3% reduction). The average difference in the Hct value (%) from spring to summer was significantly smaller (*p* < 0.01) in the control group than in the ivermectin-treated group (Fig. 3).

**Table 1:** Hematological results of the control and ivermectin-treated groups

Group	Season	RBC (M/uL) (5.0–10.0)^a^	Hct (%) (28.0–38.0)^a^	Hb (g/dL) (9.0–14.0)^a^
Control group	Spring	8.9 ± 0.1	34.4 ± 0.5	11.7 ± 0.2
	Summer	7.9 ± 0.2	30.9 ± 0.6	11.0 ± 0.2
	Difference	1.0 ± 0.1**	3.5 ± 0.4**	0.8 ± 0.1
Ivermectin-treated group	Spring	8.6 ± 0.1	31.9 ± 0.4	11.4 ± 0.1
	Summer	7.8 ± 0.1	30.2 ± 0.3	10.7 ± 0.1
	Difference	0.8 ± 0.1 **	1.8 ± 0.3**	0.7 ± 0.1**

The difference represents the subtraction of summer results from spring results

Data are presented as the mean ± SEM. *P* values were obtained by Shapiro-Wilk test (*P < 0.05 and **P < 0.01)

^a^The reference range

## Discussion

The occurrence of tick-borne diseases may have adverse effects in cattle, necessitating the development of methods to prevent these diseases in grazing cattle. In the present study, the efficacy of ivermectin in controlling *T. orientalis* infection and associated hematological findings were evaluated. Significant differences in *T. orientalis* infection and the RBC profile were observed between the control and ivermectin-treated groups when comparing the values before and after grazing. The results of this study showed that ivermectin was effective against *T. orientalis* infection and RBC hemolysis.

*T. orientalis* consists of 11 MPSP genotypes, including types 1–8 and N-1 to N-3 [10]. Phylogenetic analysis based on the MPSP sequences revealed the presence of three genotypes (types 1, 2, and 7). Of the three MPSP genotypes identified in this study, type 1 was detected most frequently on this farm. However, this finding is inconsistent with a previous study by our group showing that type 2 was the most common type in cattle in the ROK [10]. This suggests potential differences in the regional distribution of *T. orientalis*. The MPSP sequences of the three *T. orientalis* genotypes identified in this study were closely related to those previously identified in the ROK and Japan. Based on genotypic differences, type 2 was closely related to type 7 and was different from type 1 (Fig. 1). There is no report of type 7 in cattle in the ROK; thus, it is difficult to compare genetic differences. Nevertheless, the results showed genetic variations within type 2 in the ROK compared with type 1. Further studies are needed to investigate the association between each type and clinical signs.

In this study, tick infestation in cattle after ivermectin administration was not specifically evaluated because some ticks have been removed by the farm manager. This may be explained the lack of blood-sucking ticks on the cattle at the time of blood collection. The ticks examined on the grassland around the pasture were *H. longicornis*, which was the only species of ticks found in this study. We cannot make a definite conclusion about the effectiveness of ivermectin for tick infestation; however, it may potentially reduce the number of ticks.

According to the results of the current study, the infection rate of *T. orientalis* was increased in both the control and ivermectin-treated groups during grazing; however, the increase in the infection rate was slightly lower in the ivermectin-treated group. In this study, ivermectin was used as a pour-on formulation for the cattle and not as an injection prior to grazing. This may explain the observed *T. orientalis* infection in this group. The mechanism of action of ivermectin appears to vary among organisms. Ivermectin inhibits signal transmission at the neuromuscular junction of some arthropods by stimulating the release of the inhibitory neurotransmitter γ-aminobutyric acid from presynaptic nerve terminals [21]. Moreover, ivermectin suppresses the engorgement, moulting, and reproduction of various ticks, such as *Amblyomma hebraeum*, *Boophilus microplus*, and *Ixodid* ticks [22]. However, the effects of ivermectin have not been evaluated for *H. longicornis*, which is the most prevalent tick in the ROK. In this study, the infection rate of *T. orientalis* was 3 times higher in the control group than in the ivermectin-treated group. The results of this study indicated that ivermectin had protective effects against *T. orientalis* infection. Therefore, further studies are needed to compare *T. orientalis* infection rates in grazing cattle after treatment with ivermectin at different doses using different methods of application (pour or injection).

In this study, RBC parameters (RBC count, Hct value, and Hb concentration) were decreased during grazing in both the control and ivermectin-treated groups, which is consistent with the findings of a previous study [13]. Tick biting may occur during the grazing of cattle, which could result in the transmission of tick-borne diseases via hemoparasites and subsequent hemolysis. Interestingly, the difference in Hct values was the greatest, with a 2-fold reduction in the control group compared with the ivermectin-treated group, indicating that ivermectin had protective effects against hemolysis in cattle during grazing. Although the mechanisms by which ivermectin affects only Hct were not evaluated in the current study, a previous study showed that ivermectin restored reduced Hct levels to normal levels in experimentally infected lambs with *Haemonchus contortus* [23]. This may be associated with indirect protection against ectoparasites considering that the vectors of several hemoparasites can cause hemolysis in the host [13, 24]. Ivermectin is a semi-synthetic anti-parasitic drug that is used to control intestinal nematodes and ticks that affect cattle [16, 21, 23, 25]. Ivermectin is highly effective for treating endoparasite infection; however, its effects on ectoparasites in cattle are unknown. The results of this study indicated that ivermectin might be effective for preventing RBC hemolysis caused by hemoparasites. Therefore, ivermectin could be used as an anti-ectoparasitic agent. Further studies are required to assess the effects of ivermectin on other blood parameters in host animals.

## Conclusion

In this study, ivermectin was found to be effective for controlling *T. orientalis* infection and RBC hemolysis in grazing cattle. Ivermectin could prevent tick-borne diseases and related clinical symptoms, thus reducing the disadvantages associated with raising cattle on pastures.

## Methods

### Ethics statement

All animal procedures were carried out according to ethical guidelines for the use of animal samples, as approved by Chonbuk National University (Institutional Animal Care and Use Committee Decision No. CBU 2016–00026). All procedures and possible consequences were explained to the managers of the surveyed farm, and written consent was obtained.

### Experimental animals

A cattle farm, located at Jiri Mountain in the ROK, was selected for this study. This farm was well maintained and was consigned by nearby farms to raise Holstein cattle until pregnancy. The cattle were raised on a pasture near the mountain area from spring to autumn. A total of 68 cattle with no prior experience of grazing on this farm were divided into two groups according to treatment with ivermectin (IMEC POUR-ON; ECO Animal Health Ltd., London, United Kingdom); the control group (*n* = 17) was not treated with ivermectin before grazing, whereas the experimental group (*n* = 51) received ivermectin treatment. Ivermectin was poured along the midline in a narrow strip extending from the withers to the tailhead at a dose of 1 mL (5 mg of ivermectin)/10 kg body weight once before grazing (as per the manufacturer’s instructions).

### Blood collection and hematological examination

Blood (5 mL) was collected from the jugular veins of 136 cattle (68 cattle during spring [before grazing] and 68 cattle during summer [during grazing]; 17 in the control group and 51 in the experimental group). Each blood sample was stored in EDTA-supplemented tubes at 4°C and immediately transported to the laboratory. Hematological examination of the RBC profile (RBC count, hemoglobin [Hb] value, hematocrit [Hct] concentration) was performed using an automatic blood analyzer (IDEXX ProCyte Dx; IDEXX Laboratories, Westbrook, ME, USA).

### PCR and sequencing

Genomic DNA was extracted from 200 μL of each blood sample using the DNeasy Blood Kit (Qiagen Inc., Valencia, CA, USA), according to the manufacturer’s instructions. Tick-borne pathogens including *Anaplasma* spp., *Babesia* spp., *Ehrlichia* spp., *Rickettsia* spp., and *T. orientalis* were screened (Table 2) under the following cycling conditions: 95 °C for 15 min, followed by 40 cycles of 95 °C for 10 s, 58 °C for 30 s, and 72 °C for 30 s, and a final extension at 72 °C for 5 min. In all experiments, a negative control was included in the PCR assay. The amplicons were run on a 1.5% agarose gel and visualized following ethidium bromide staining. The PCR products were purified using the AccuPrep® PCR Purification Kit (Bioneer, Daejeon, ROK) and directly sequenced (Macrogen, Inc., Seoul, ROK).

**Table 2:** Primer sequences and PCR conditions used for the detection of tick-borne pathogens in this study

Tick-borne pathogen	Specific genes	Sequence (5′ to 3′)	Annealing temperature (°C)	Amplicon size (bp)	Reference
*Anaplasma* spp.	16S rRNA	TACCTCTGTGTTGTAGCTAACGC CTTGCGACATTGCAACCTATTGT	58	429	[26]
*Babesia* spp.	18S rRNA	GTTTCTGMCCCATCAGCTTGAC CAAGACAAAAGTCTGCTTGAAAC	61	420–440	[27]
*Ehrlichia* spp.	16S rRNA	CGGAATTCCTAGTGTAGAGG AGGAGGGATACGACCTTCAT	58	340	[26]
*Rickettsia* spp.	16S rRNA	TAGGGGATGATGGAATTCCTA CCCCCGTCA ATTCCTTTGAG	58	252	[26]
*Theileria orientalis*	MPSP*	CACGCTATGTTGTCCAAGAG TGTGAGACTCAATGCGCCTA	55	830	[28]

*MPSP: major piroplasm surface protein

### Phylogenetic analysis

DNA sequencing data were analyzed by the Basic Local Alignment Search Tool (BLAST) in the National Center for Biotechnology Information (NCBI) database to determine the homology of *T. orientalis* major piroplasm surface protein (MPSP) genes. The sequences were aligned using ClustalX program. A phylogenetic tree was constructed based on the nucleotide alignments using the neighbor-joining method [29]. Bootstrap analysis was conducted with 1000 replicates using MEGA version 6 [30]. The 8 sequences obtained in this study were deposited in the GenBank database under the accession numbers MN128425-MN128430 and KY018576- KY018577.

### Statistical analysis

All statistical analyses were performed using the SPSS 24.0 software package (SPSS, Chicago, IL, USA). Hematological results are expressed as the mean ± standard error of the mean (SEM). Seasonal changes in blood test results were compared between two groups using two-tailed independent *t*-tests depending on the results of normality tests (Shapiro-Wilk test). Differences in the seasonal changes in blood test results between the two groups were also analyzed using two-tailed independent *t*-tests depending on the results of normality tests (Shapiro-Wilk tests). All graphical procedures were performed using GraphPad Prism 6 for Windows (GraphPad Software Inc., San Diego, CA, USA). Differences with *p* values of less than 0.05 were considered significant.

## Data Availability

All data generated or analyzed during this study are included in the article.

## Abbreviations

*H. longicornis*: *Haemaphysalis longicornis*
Hb: hemoglobin
Hct: hematocrit
MPSP: major piroplasm surface protein
PCR: polymerase chain reaction
RBC: red blood cell
ROK: Republic of Korea
SEM: standard error of the mean
*T. orientalis*: *Theileria orientalis*
